# Mud and Muddle

**Published:** 1892-04-09

**Authors:** 


### Passing Topics.

### MUD AND MUDDLE.

What a great and good thing it would be if only there were some way of preventing any but properly-qualified people from giving practical effect to their opinions in matters concerning the community. The discomfiture of amateurism would be a triumph of reason of which the century might well be proud. We are not thinking of amateur efforts in art and literature. They affect nobody but the artist and his friends, if he retain any. We are not thinking even of the amateur philanthropist or politician, with his probably impossible programme. The real amateur, moreover, is worthy of all respect, if not of admiration, for we take him to be one who puts his soul into all he attempts, never scamps anything, but strives honestly to turn out the very best work of which he is capable. All honour to the true amateurs, even though they never get on good terms with their ambitions. We have in mind now those mischievous people who commit themselves to ideas and actions without ever having taken the trouble to justify them to themselves, people who use words they cannot spell, and jump to conclusions they know no other way of reaching.

Especially is it to be deplored when this sort of person proceeds to turn his attention to parish matters, for so great is the dearth of capable people who are willing to serve on parochial committees that if persistent, as he usually is, he will certainly obtain, sooner or later, the coveted seat at the vestry, the local board of health, or some other body charged with duties which affect in no small degree the comfort and well-being of his neighbours. If we were pressed to formulate the indications which are characteristic of the typical local board, we should begin with the water-cart. Slop, mud, and general discomfort seem to embody the chief aspirations of the authorities. Witness our streets any day these three months. When frost and snow came together in February and March, applications of salt were liberally made, and in certain districts, miles of pavement were for days together deep in the most abominable, and to the poorly shod, most deadly mixture the wit of vestrymen could devise. Similarly, directly the fine weather set in on the eve of the current month, the vestrymen, as if in honour of their festival, sent out the water carts, and turned the streets, dry and clean almost for the first time since the year before last, into rivers of slosh.

Now clearly, if vestrymen and members of local boards were compelled to qualify for the positions they occupy, we might hope for better things than these. A fall of snow is not so unprecedented an occurrence that anybody, least of all those who pretend to govern, is justified in ignorance of how to deal with it, and the wickedness—we write feelingly—of doing away with one of the chief advantages of fine weather, a clean roadway, by creating mud artificially, ought to be rendered penal. Deluging a street even when it is really dusty is a barbarous proceeding; it ought to be sprinkled, and as often as necessary. One can bear mud in wet weather with something like resignation, but to be splashed from head to foot by passing vehicles when there is no necessity, and to sink ankle deep in mire when one crosses a street are trying ordeals, while to undergo a purely gratuitous process of incrustation in a hot sun is beyond the patience of any being but an alligator or a hippopotamus. Agricola.

